# Functionalized porous silica&maghemite core-shell nanoparticles for applications in medicine: design, synthesis, and immunotoxicity

**DOI:** 10.3325/cmj.2016.57.165

**Published:** 2016-04

**Authors:** Beata A. Zasońska, Aurélia Líšková, Miroslava Kuricová, Jana Tulinská, Ognen Pop-Georgievski, Fedor Čiampor, Ivo Vávra, Mária Dušinská, Silvia Ilavská, Mira Horváthová, Daniel Horák

**Affiliations:** 1 Institute of Macromolecular Chemistry, Academy of Sciences of the Czech Republic, Prague, Czech Republic; 2 Medical Faculty, Slovak Medical University, Bratislava, Slovakia; 3 Institute of Virology, Slovak Academy of Sciences, Bratislava, Slovakia; 4 Institute of Electrical Engineering, Slovak Academy of Sciences, Bratislava, Slovakia; 5 Health Effects Laboratory, Department of Environmental Chemistry, NILU-Norwegian Institute for Air Research, Kjeller, Norway

## Abstract

**Aim:**

To determine cytotoxicity and effect of silica-coated magnetic nanoparticles (MNPs) on immune response, in particular lymphocyte proliferative activity, phagocytic activity, and leukocyte respiratory burst and *in vitro* production of interleukin-6 (IL-6) and 8 (IL-8), interferon-gamma (IFN-γ), tumor necrosis factor-alpha (TNF-α), and granulocyte macrophage colony stimulating factor (GM-CSF).

**Methods:**

Maghemite was prepared by coprecipitation of iron salts with ammonia, oxidation with NaOCl and modified by tetramethyl orthosilicate and aminosilanes. Particles were characterized by transmission electron microscopy (TEM), dynamic light scattering (DLS), Fourier-transform infrared (FTIR), and X-ray photoelectron spectroscopy (XPS). Cytotoxicity and lymphocyte proliferative activity were assessed using [^3^H]-thymidine incorporation into DNA of proliferating human peripheral blood cells. Phagocytic activity and leukocyte respiratory burst were measured by flow cytometry; cytokine levels in cell supernatants were determined by ELISA.

**Results:**

γ-Fe_2_O_3_&SiO_2_-NH_2_ MNPs were 13 nm in size. According to TEM, they were localized in the cell cytoplasm and extracellular space. Neither cytotoxic effect nor significant differences in T-lymphocyte and T-dependent B-cell proliferative response were found at particle concentrations 0.12-75 μg/cm^2^ after 24, 48, and 72 h incubation. Significantly increased production of IL-6 and 8, and GM-CSF cytokines was observed in the cells treated with 3, 15, and 75 µg of particles/cm^2^ for 48 h and stimulated with pokeweed mitogen (PHA). No significant changes in TNF-α and IFN-γ production were observed. MNPs did not affect phagocytic activity of monocytes and granulocytes when added to cells for 24 and 48 h. Phagocytic respiratory burst was significantly enhanced in the cultures exposed to 75 µg MNPs/cm^2^ for 48 h.

**Conclusions:**

The cytotoxicity and *in vitro* immunotoxicity were found to be minimal in the newly developed porous core-shell γ-Fe_2_O_3_&SiO_2_-NH_2_ magnetic nanoparticles.

Superparamagnetic nanoparticles containing both nonporous and porous shells have been intensively investigated in recent years with respect to their potential applications in separation of molecules (1), sensors (2), as contrast agents in magnetic resonance imaging (MRI) (3), as well as carriers of drugs and biomolecules (4) for diagnostic (5) and therapeutic purposes (6).

Magnetic nanoparticles (MNPs), such as magnetite (Fe_3_O_4_) or maghemite (γ-Fe_2_O_3_), received considerable attention for their small size, spherical shape, strong magnetic properties enabling targeting to a specific organ using an external magnetic field (7), and rather low toxicity (8). However, neat (uncoated) iron oxide particles showed toxicity at high doses, tendency to aggregate, and high nonspecific adsorption of biomolecules, which is inacceptable for *in vitro* or *in vivo* medical applications (9). This can be avoided by surface modifications, such as grafting (coating) and/or encapsulation of MNPs by polymers, which are the most frequently used materials to improve biocompatibility and stability of the iron oxide nanoparticles in aqueous media. Some examples of polymers include dextran, poly(vinyl alcohol) (10), chitosan (11), poly(methyl methacrylate) (12), poly(ethylene glycol) (13), or poly(L-lysine). One of the universal agents to modify the iron oxide surface is also silica (14). This chemically inert inorganic material has a lot of advantages for biomedical applications compared to other materials. It is porous, biocompatible, modifiable with various functional agents, and has no toxicity at moderate concentrations (15); however silica dust is harmful if inhaled, and induces silicosis. Silica derivatives can introduce functional groups, eg, NH_2_, COOH, SH, on the iron oxide surface to enable immobilization of target biomolecules. Mesoporous silica nanoparticles are thus attractive for drug loading and controlled release (16).

The aim of this research was to synthesize γ-Fe_2_O_3_, γ-Fe_2_O_3_&SiO_2_, and γ-Fe_2_O_3_&SiO_2_-NH_2_ MNPs and thoroughly characterize them. We also aimed to investigate *in vitro* cytotoxicity and immunotoxicity of the γ-Fe_2_O_3_&SiO_2_-NH_2_ nanoparticles.

## Materials and methods

### Materials

FeCl_2_ · 4H_2_O and FeCl_3_ · 6H_2_O were purchased from Fluka (Buchs, Switzerland). Sodium hypochlorite solution (5 wt.%) was from Bochemie (Bohumín, Czech Republic). Tetramethyl orthosilicate (TMOS), (3-aminopropyl)triethoxysilane (APTES), (3-aminopropyl)dimethylethoxysilane (APDMES), RPMI-1640 medium, phytohemagglutinin (PHA), concanavalin A (Con A), pokeweed mitogen (PWM), L-glutamine, and hydroethidine were purchased from Sigma-Aldrich (Steinheim, Germany). Anti-CD3 monoclonal antibody was purchased from Beckman Coulter and fetal calf serum (FCS) from PAA. Fluorescein-labeled *Staphylococcus aureus* bacteria and opsonizing reagent were purchased from Molecular Probes (Eugene, OR, USA). Cationic surfactant, cetyltrimethylammonium bromide (CTAB) was purchased from Lachema (Brno, Czech Republic). Ammonium hydroxide solution (25%) and ethanol were purchased from Lach-Ner (Neratovice, Czech Republic). Gentamycin was purchased from Sandoz (Bratislava, Slovakia), cyclophosphamide (Cyf) from Baxter (Deerfield, IL, USA), and [^3^H]-thymidine from Moravek Biochemicals (Brea, CA, USA). Scintillation fluid was purchased from Perkin Elmer (Waltham, MA, USA). Ultrapure Q-water ultrafiltered on a Milli-Q Gradient A10 system (Millipore, Molsheim, France) was used for preparation of the solutions. ELISA sets were purchased from Affymetrix e-Biosciences.

### Preparation of γ-Fe_2_O_3_ nanoparticles

Iron oxide nanoparticles were prepared from iron(III) chloride and iron(II) chloride by a standard procedure (17). Aqueous 0.2 M FeCl_3_ (100 mL), 0.2 M FeCl_2_ (50 mL), and 0.5 M NH_4_OH (100 mL) solutions were sonicated (Sonicator W-385; Heat Systems-Ultrasonics; Farmingdale, USA) for 5 min. The mixture was added to 3.3 wt.% NH_4_OH (460 mL) and stirred at 23°C for 1 h (200 rpm). Magnetite was magnetically separated and washed by Q-water until peptization. Then, 5 wt.% sodium hypochlorite solution (16 mL) was added and the system sonicated for 5 min. The precipitate was magnetically separated and repeatedly washed with Q-water until formation of γ-Fe_2_O_3_ colloid.

### Synthesis of porous core-shell γ-Fe_2_O_3_&SiO_2_-NH_2_ nanoparticles

First, silica shell was introduced on the particles by modification of Stöber method using hydrolysis and condensation of TMOS (18). In a typical experiment, ethanol (60 mL), water (3 mL), and 25 wt.% ammonia (0.3 mL) were mixed with γ-Fe_2_O_3_ (150 mg) for 5 min. TMOS (0.1 mL) was added and the mixture stirred (400 rpm) at 50°C for 16 h. The resulting γ-Fe_2_O_3_&SiO_2_ colloid was three times washed with ethanol using magnetic separation.

**Figure 1:**
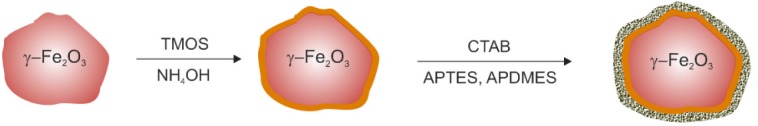
Scheme of silanization of γ-Fe_2_O_3_ with tetramethyl orthosilicate (TMOS) and (3-aminopropyl)triethoxysilane/(3-aminopropyl)dimethylethoxysilane (APTES/APDMES) to introduce NH_2_ groups on the particle surface.

The second (porous) shell containing amino groups on the surface was introduced by modification of γ-Fe_2_O_3_&SiO_2_ nanoparticles with APTES/APDMES mixture (Figure 1). Briefly, γ-Fe_2_O_3_&SiO_2_ nanoparticles were dispersed in ethanol (60 mL) and CTAB (0.15 g), APTES (0.05 mL), APDMES (0.05 mL), and water (1 mL) were added. The mixture was stirred using an anchor-type stirrer (400 rpm) at 50°C for 24 h. After completion of the reaction, the resulting porous γ-Fe_2_O_3_&SiO_2_-NH_2_ particles were washed using magnetic separation (5 times with ethanol and 3 times with water) and centrifugation (3 times).

### Characterization of nanoparticles

The synthesized nanoparticles were characterized by a Tecnai Spirit G2 transmission electron microscope (TEM; FEI) and number- (*D*_n_) and weight-average particle diameter (*D*_w_) and polydispersity index (PDI = *D*_w_/*D*_n_) were calculated by analyzing ca. 400 particles. The hydrodynamic particle size (*D*_h_) of γ-Fe_2_O_3_ and γ-Fe_2_O_3_&SiO_2_ and polydispersity PI (0-1) was determined by dynamic light scattering (DLS) using a Zetasizer Nano-ZS Model ZEN3600 instrument (Malvern Instruments; Malvern, UK). Fourier-transform infrared (FTIR) spectra were recorded in an attenuated total reflection (ATR) mode using a Thermo Nicolet NEXUS 870 FT-IR Spectrometer (Madison, WI, USA). Specific surface area (*S*_BET_) of the nanoparticles was determined by dynamic nitrogen adsorption using a Gemini VII 2390 Analyzer (Micromeritics; Norcross, GA, USA). X-ray photoelectron spectroscopy (XPS) was carried out with a K-Alpha + spectrometer (ThermoFisher Scientific) equipped with a micro-focused monochromated Al Kα X-ray source (400 µm spot size). The kinetic energy of the electrons was measured using a 180° hemispherical energy analyzer operated in the constant energy mode at 200 and 50 eV pass energy for survey and high resolution spectra, respectively. Data acquisition and processing were performed using Thermo Advantage software. The XPS spectra were fitted with one or more Voigt profiles (binding energy BE, uncertainty ±0.2 eV). The analyzer transmission function, Scofield sensitivity factors, and effective attenuation lengths for photoelectrons were calculated using the standard TPP-2M formalism. All spectra were referenced to the C 1s peak of hydrocarbons at 285 eV of BE controlled by means of the well-known photoelectron peaks of metallic Cu, Ag, and Au.

### Participants

Ten healthy male volunteers participating in the study signed an informed consent approved by the Ethics Committee of the Slovak Medical University in Bratislava. Blood was collected by venepuncture using heparinized tubes.

### Preparation of MNPs for cell treatment

Porous γ-Fe_2_O_3_&SiO_2_-NH_2_ core-shell nanoparticles were vortex-shaken in the tubes for a few minutes before use and diluted with the RPMI 1640 medium containing 10% FCS to obtain a stock solution (75 µg/cm^2^). Serial dilutions of this solution in the cell culture medium were prepared to obtain the full concentration range of MNP dispersions: 0.12, 0.6, 3, 15, and 75 μg/cm^2^ corresponding to 0.17, 0.85, 4.24, 21.2, and 106 μg of particles/mL, respectively. In all assays, MNPs were added to the cell cultures in a volume of 25 µL.

### Interaction of nanoparticles with peripheral blood cells according to TEM

Human heparinized whole blood (diluted 1:1 with phosphate buffered saline, PBS) was layered on Lymphosep (MP Biomedicals) and centrifuged for 30 min (700 *g*). Mononuclear cells were collected from interphase and washed in PBS and Roswell Park Memorial Institute medium (RPMI 1640) with 10% fetal calf serum (FCS). Cells were adjusted to 2.5 × 10^6^ cells/mL in RPMI, 10% FCS, and pipetted in sixplicates in a volume of 180 µL to the microplates. MNPs in concentrations 0.12, 3, and 75 µg/cm^2^ were added in a volume of 20 μL. The plates were incubated at 37°C for 24 h under 5% CO_2_ atmosphere. Then the cells were two times washed with saline, centrifuged, saline was decanted, and the cells were fixed with 2.5% glutaraldehyde in PBS (pH 7.2) at room temperature for 60 min. Subsequently, the cells were washed with PBS and dehydrated with increasing concentration of ethanol (30, 50, 70, 90, 2 × 100%) in the solution. The cell pellet was embedded in London Resin White (Polysciences; Warrington, PA, USA) and polymerized at 60°C for 24 h. Ultrathin sections were prepared using LKB ultramicrotome and captured on EM mesh without the support membrane without staining. Ultrathin sections were evaluated using a JEOL 1200 EX TEM microscope with 100 kV accelerating voltage.

### Assessment of cytotoxicity and immunotoxicity of γ-Fe_2_O_3_&SiO_2_-NH_2_ nanoparticles

*Cytotoxicity and proliferative activity of lymphocytes.* Cytotoxicity and proliferative activity of lymphocytes were assessed by ^3^H-thymidine incorporation into DNA of proliferating cells using a liquid scintillation. Human heparinized whole blood (150 μL) diluted 1:15 in complete RPMI 1640 medium containing 10% FCS, L-glutamine, and gentamycin was dispensed in triplicate wells of a 96-well microtiter culture plate under sterile conditions. Mitogens were added to obtain the following final concentrations: concanavalin A (Con A) (25 μg/mL), phytohemagglutinin (PHA) (25 μg/mL), pokeweed mitogen (PWM) (2.5 μg/mL), and antigen CD3 (3 μg/mL). γ-Fe_2_O_3_&SiO_2_-NH_2_ nanoparticles (25 μL) were added in different exposure intervals (24, 48, and 72 h) before the end of whole 72 h incubation period. Cyf (40 mg/mL) was used as a suppressive control in unstimulated and PHA-stimulated cultures. The plates were incubated at 37°C for 48 h under 5% CO_2_ atmosphere; then wells were pulsed with 1 μCi [^3^H]-thymidine diluted in medium (20 μL) and incubated at 37°C for additional 24 h. After the whole 72 h incubation period, cell cultures were harvested onto glass filter paper, which was placed into a scintillation fluid. Radioactivity was measured using a Microbeta 2 scintillation counter (Perkin Elmer). Additionally, interference of the nanoparticles with the assay was tested, when the particles were added to the control cultures (unstimulated or stimulated with mitogens and antigen) few minutes before harvesting of the cells. Counts per minute (cpm)/per culture were calculated in triplicate for each variable.

*In vitro production of interlekin-6 (IL-6), interleukin-8 (IL-8), interferon-gamma (IFN-γ), tumor necrosis factor-alpha (TNF-α), and granulocyte macrophage colony-stimulating factor (GM-CSF).* Human heparinized blood diluted in complete RPMI-1640 medium (10% FCS) with PHA mitogen and the nanoparticles were cultivated for 72 h as described above. The nanoparticles were added 24 h after the beginning of the 72 h incubation period. After the incubation, cell culture supernatants were removed and frozen at -70°C. The levels of cytokines IL-6, IL-8, IFN-γ, TNF-α, and GM-CSF in the cell supernatants were analyzed by ELISA according to the manufacturer’s procedure.

*Phagocytic activity and respiratory burst of leukocytes.* Phagocytic activity and respiratory burst of leukocytes were examined by an Epics XL flow cytometer. Briefly, 150 μL of human heparinized blood (diluted 1:1 in RPMI-1640 consisting of 10% FCS, L-glutamine, and gentamycin) was distributed into wells of 96-well culture microplate under a sterile conditions, γ-Fe_2_O_3_&SiO_2_-NH_2_ nanoparticles (25 μL) were added, and the mixture incubated for 24 and 48 h. Cyf (5 mg/mL) was used as a suppressive control. After the incubation, blood (30 μL) from each well was pipetted into the tube and hydroethidine solution (10 μL) was added. First, the samples were incubated at 37°C for 15 min and 3 μL of fluorescein-labeled *Staphylococcus aureus* bacteria (1.4 × 10^6^ per test) was added. Second, all tubes were incubated at 37°C for another 15 min, the samples were put on an ice, and cold lysis solution (700 μL) was added. In the control tubes, *S. aureus* was added after the lysis solution. The samples were tested in duplicates and analyzed by flow cytometry within 30 min as before. Phagocytic activity of granulocytes and monocytes and respiratory burst of phagocytes were measured. Interference of particles with the assay was tested by measuring the same control tubes without particles and few seconds after addition of particles.

### Statistical analysis

SPSS 16.0 software (Chicago, IL, USA) was used for statistical analysis. Triplicates or duplicates from each human subject were averaged and used as a single value for statistical analysis. Normality was determined by Shapiro-Wilk test. The paired-sample *t* test and the Mann-Whitney U-test (or Wilcoxon’s test) were used to estimate significant differences between groups for normally and non-normally distributed data, respectively. Data were expressed as the mean values with a standard deviation. Differences at *P* < 0.05 were considered to be statistically significant.

## Results

**Figure 2:**
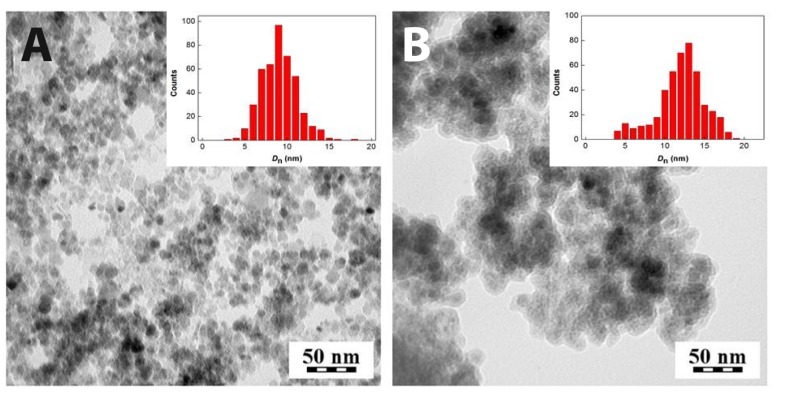
Transmission electron micrographs of (**A**) γ-Fe_2_O_3_ and (**B**) γ-Fe_2_O_3_&SiO_2_-NH_2_ nanoparticles.

Maghemite nanoparticles (γ-Fe_2_O_3_) were prepared according to the following reactions:

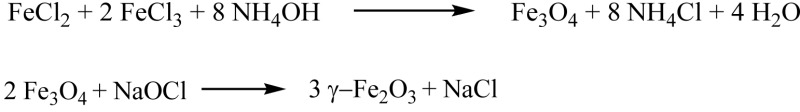

where magnetite (Fe_3_O_4_) was obtained by coprecipitation of FeCl_2_ and FeCl_3_ by ammonia, which was followed by oxidation. The final product (γ-Fe_2_O_3_) was brownish with high absolute value of ζ-potential (-41 mV) and exhibiting strong magnetization under magnetic field (19). Morphology of the resulting products was investigated by TEM, which images the dry particles (Figure 2). However, drying of aqueous colloids always leads to an aggregation. Nevertheless, potentially individual almost spherical particles can be distinguished and their diameters were measured for size statistics. Diameter of the γ-Fe_2_O_3_ particles was 9 nm and the particle size distribution was rather narrow (PDI = 1.21). After hydrolysis and condensation of TMOS, APTES and APDMES, size of the γ-Fe_2_O_3_&SiO_2_-NH_2_ particles increased to 13 nm (Table 1) due to formation of 2 nm-thick silica shell seen as a low-contrast layer (Figure 2B). Silica shell increased also the hydrodynamic particle diameter from 176 (γ-Fe_2_O_3_) to 474 nm (γ-Fe_2_O_3_&SiO_2_-NH_2_), while the PI increased from ∼ 0.1 to 0.2. The reason for such large differences between *D*_n_ and *D*_h_ is that the DLS provides information about the particle dimers and clusters in water, while TEM shows individual particles in the dry state. DLS measurements of γ-Fe_2_O_3_&SiO_2_-NH_2_ nanoparticles incubated with serum at 37.7°C for 24 h showed even larger hydrodynamic size ~ 600 nm and size distribution PI ~ 0.3-0.4. The γ-Fe_2_O_3_&SiO_2_-NH_2_ particle colloids were stable; a tendency to aggregation was observed up to few months of storage. The results from dynamic adsorption of nitrogen are presented in Table 1. The specific surface area increased from 65.9 (neat γ-Fe_2_O_3_) to 206 m^2^/g for γ-Fe_2_O_3_&SiO_2_-NH_2_ documenting thus porous character of the silica shell. Porosity was obviously induced by CTAB present in the silanization mixture; after completion of the reaction, CTAB was removed from the silica by washing with ethanol and water using magnetic separation and centrifugation.

**Table 1:** Properties of the developed nanoparticles*

Nanoparticles	*D*_n_ (nm)	PDI	*D*_h_ (nm)	PI	Mixed with blood serum after incubation		*S*_BET_ (m^2^/g)
					*D*_h_ (nm)	PI	
γ-Fe_2_O_3_	9	1.21	176	0.09	623	0.43	66
γ-Fe_2_O_3_&SiO_2_	10	1.28	260	0.08	-	-	167
γ-Fe_2_O_3_&SiO_2_-NH_2_	13	1.34	474	0.2	598	0.34	206

*D_n_ – number average particle diameter (TEM), PDI – polydispersity index (TEM), *D*_h_ – hydrodynamic diameter (DLS), PI – polydispersity (DLS), *S*_BET_ – specific surface area.

**Figure 3:**
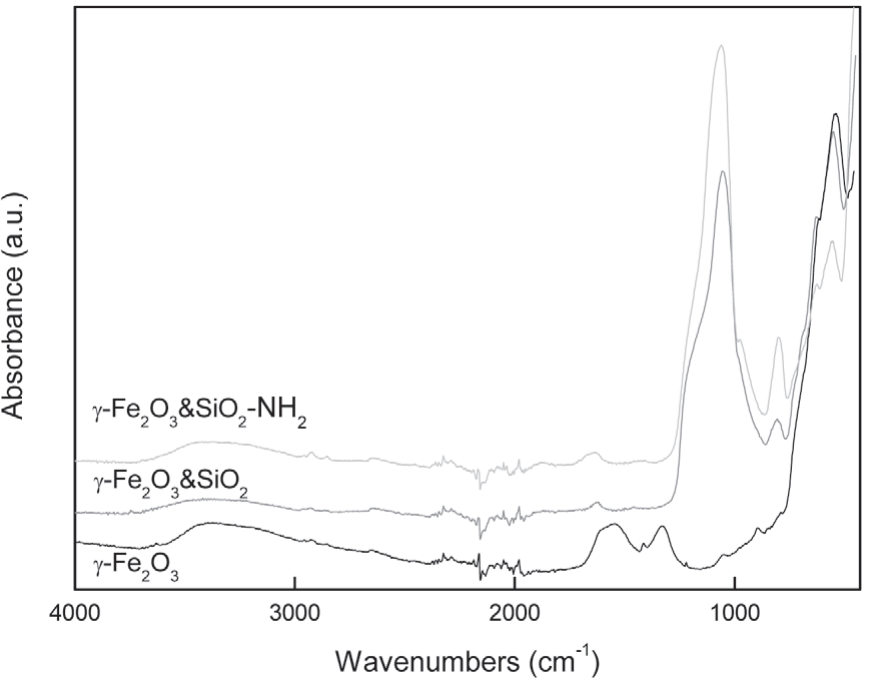
Attenuated total reflection Fourier-transform infrared spectra of γ-Fe_2_O_3_, γ-Fe_2_O_3_&SiO_2_, and γ-Fe_2_O_3_&SiO_2_-NH_2_ nanoparticles.

FTIR analysis confirmed successful coating of the neat γ-Fe_2_O_3_ particles with the silica modification agents (Figure 3). The spectrum of the γ-Fe_2_O_3_ showed absorption bands at 3372, 1558, 1418, 896, and 542 cm^-1^. The range of 3600-3100 cm^-1^ was attributed to antisymmetric and symmetric O–H stretching, which can suggest the presence of adsorbed water on the particle surface (20). Wide peak at 542 cm^-1^ is characteristic for the metal oxygen stretching vibrations. After the first modification of γ-Fe_2_O_3_ nanoparticles with TMOS, the spectrum of γ-Fe_2_O_3_&SiO_2_ confirmed the presence of SiO_2_ shell around the iron oxide cores (Figure 3B). Shift of the maximum of Si-O-C asymmetric stretching vibration at ∼ 1054 cm^–1^ was ascribed to the interaction of SiO_2_ with γ-Fe_2_O_3_. When the γ-Fe_2_O_3_&SiO_2_ was modified with APTES, the spectrum of γ-Fe_2_O_3_&SiO_2_-NH_2_ nanoparticles was shifted and the presence of NH_2_ groups was well visible at 1638 and 1472 cm^–1^; N-H stretching vibration at ~ 3300 cm^–1^ was only slightly noticeable. The peaks at 1480, 1450, and 1395 cm^–1^ (CH_3_ deformation vibrations) were present in spectra of both γ-Fe_2_O_3_&SiO_2_ and γ-Fe_2_O_3_&SiO_2_-NH_2_ particles.

### X-ray photoelectron spectroscopy (XPS)

**Figure 4:**
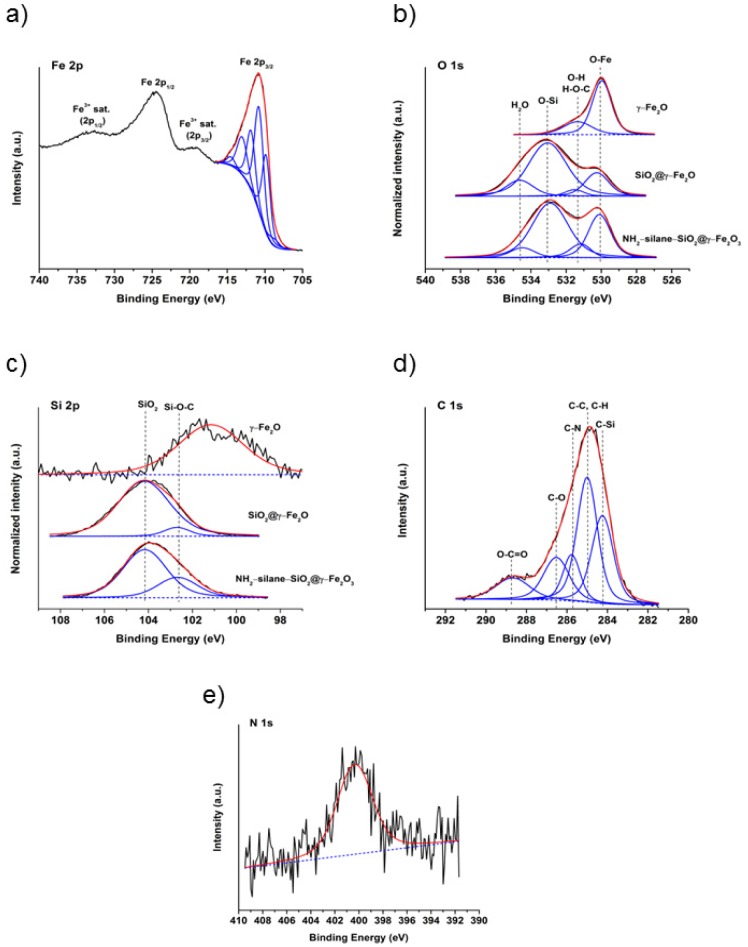
Measured high resolution (**A**) Fe 2p, (**B**) O 1s, (**C**) Si 2p, (**D**) C 1s, and (**E**) N 1s X-ray photoelectron spectra (XPS; black) fitted (red) by resolving the individual components (blue). Simulated Fe 2p_3/2_ spectrum was obtained by considering Gupta-Sen multiplets and satellite peaks of high and low binding energy (26). O 1s high-resolution XPS spectra of neat γ-Fe_2_O_3_, γ-Fe_2_O_3_&SiO_2_, and γ-Fe_2_O_3_&SiO_2_-NH_2_ nanoparticles showed the presence of surface oxides, hydroxyls, and adsorbed water. Si 2p spectra confirmed the formation of SiO_2_ and SiO_2_-NH_2_ shell on the γ-Fe_2_O_3_ surface. The presence of amino groups on the γ-Fe_2_O_3_&SiO_2_-NH_2_ surface was further verified by the detailed analysis of the C 1s and N 1s spectra.

The XPS results confirmed that the neat γ-Fe_2_O_3_ nanoparticles contained Fe and O with Fe/O ratio = 0.55, which is close to the theoretically expected value of 0.67 (Table 2; Figure 4). The Fe 2p spectrum showed two main peaks at 710.8 and 724.4 eV corresponding to Fe 2p_3/2_ and Fe 2p_1/2_, respectively (Figure 4A). These peaks were accompanied by weakly pronounced satellite peaks. Importantly, the Fe 2p_3/2_ satellite peak (719.0 eV) appeared at separation energy of 8.2 eV, thus verifying the presence of Fe^3+^ species. The absence of satellite peaks at 716.0 eV characteristic for Fe^2+^ confirmed the absence of Fe_3_O_4_ in the nanoparticles. The O 1s spectrum of the neat nanoparticles showed two contributions at 530.0 and 531.3 eV arising from γ-Fe_2_O_3_ lattice oxygen and surface hydroxyls (Figure 4B). The latter band had contributions from the adventitious organic carbon contaminants originating from alcohol contaminants as further evidenced by the peak at 285.0 eV. The Si 2p spectrum of the neat nanoparticles also showed a minute contribution of Si at 101.2 eV (Figure 4C). The low intensity of this band made the exact identification of the silicon binding state difficult.

**Table 2:** Surface atomic percentages for γ-Fe_2_O_3_, γ-Fe_2_O_3_&SiO_2_, and γ-Fe_2_O_3_&SiO_2_-NH_2_ nanoparticles determined by X-ray photoelectron spectroscopy

	Atomic concentration (%)						
Nanoparticles	Fe 2p	O 1s	C 1s	Si 2p	N 1s	Na 1s	Cl 2p
γ-Fe_2_O_3_	27.7	50.5	17.6	0.8	-	2.5	0.9
γ-Fe_2_O_3_&SiO_2_	8.1	58.1	10.2	23.6	-	-	-
γ-Fe_2_O_3_&SiO_2_-NH_2_	12.5	55.9	10.6	20.6	0.4	-	-

The formation of SiO_2_ shell on the γ-Fe_2_O_3_ nanoparticle surface was clearly confirmed by decrease of the Fe and increase of Si and O content in the γ-Fe_2_O_3_&SiO_2_ (Table 2). The O 1s and Si 2p regions were characterized by new dominating bands at 533.1 and 104.1 eV, respectively, which are typical of pure silica (Figure 4C). Concomitantly, the initially observed contributions of γ-Fe_2_O_3_ lattice oxygen and surface hydroxyls were significantly lowered.

Introduction of aminosilica (using APTES and APDMES) on the nanoparticle surface did not change the O 1s spectra. The Si 2p region of the γ-Fe_2_O_3_&SiO_2_-NH_2_ nanoparticles was characterized by a stronger contribution at 102.6 eV arising from the Si-C bonds of APTES and APDMES. Importantly, the high-resolution XPS spectrum in the C 1s and N 1s region clearly showed the silane coating containing amino terminal groups (Figure 4 D, E). The C 1s envelope of the silane layer could be resolved into contributions centered at 284.2, 285.0, 285.7, and 286.5 eV arising from C-Si, sp^3^ carbon (C-C and C-H functionalities), C-N species of amines, and C-O contribution of hydroxyls present in the non-hydrolyzed ethoxy groups of APTES, respectively. We assigned the peak at 288.7 eV to carbamates formed by reaction of amino groups with atmospheric CO_2_.

### TEM assessment of interaction of γ-Fe_2_O_3_&SiO_2_-NH_2_ nanoparticles with peripheral blood cells

**Figure 5:**
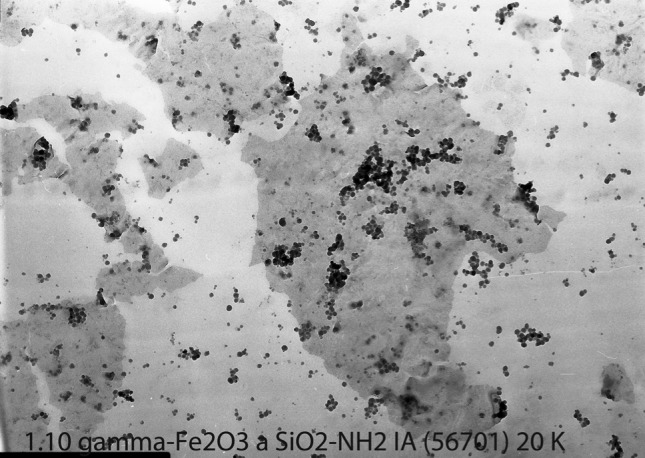
Transmission electron micrograph of ultrathin section of blood cells treated with γ-Fe_2_O_3_&SiO_2_-NH_2_ nanoparticles. They are localized within the cytoplasm of treated cells and in the extracellular space. There is no preferential localization in the cells, majority of the nanoparticles is localized on the cell surface and in cell surroundings. Magnification: 20 000 × .

TEM micrograph of ultrathin section of peripheral blood mononuclear cells treated with the γ-Fe_2_O_3_&SiO_2_-NH_2_ nanoparticles showed that they were localized within the cytoplasm and in the extracellular space (Figure 5). Microphotograph did not display any preferential localization of nanoparticles in the cells. Majority of the nanoparticles was localized on the cell surface and in cell surroundings.

### Assessment of immunotoxicity of γ-Fe_2_O_3_&SiO_2_-NH_2_ nanoparticles

*Cytotoxicity of γ-Fe_2_O_3_&SiO_2_-NH_2_ nanoparticles to peripheral blood cells and the effect of particles on proliferative activity of lymphocytes.* In these experiments, cytotoxicity and proliferative activity of lymphocytes incubated with γ-Fe_2_O_3_&SiO_2_-NH_2_ nanoparticles was assessed using ^3^H-thymidine incorporation into DNA of proliferating peripheral blood cells. Since the particles showed interference with the assay (data not shown), cultures with the nanoparticles added few minutes before cell harvesting were used as controls for statistical analysis and graphic display of the results.

**Figure 6:**
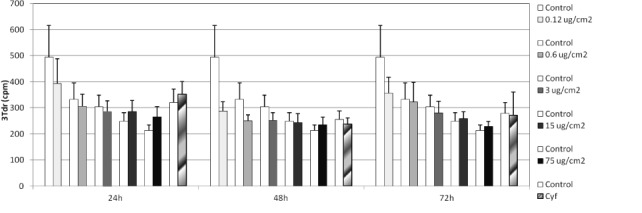
Cytotoxicity of γ-Fe_2_O_3_&SiO_2_-NH_2_ nanoparticles for human peripheral blood cells determined with [^3^H]-thymidine incorporation assay. Results are expressed as counts/min/culture (cpm). Bars indicate mean cpm group values (+ standard deviation) in non-stimulated cell cultures. Blood cultures were treated with 0 (control), 0.12, 0.6, 3, 15, and 75 μg of γ-Fe_2_O_3_&SiO_2_-NH_2_/cm^2^. The assay was performed after 24, 48, and 72 h of *in vitro* exposure of the peripheral blood cells (n = 10 human volunteers) with γ-Fe_2_O_3_&SiO_2_-NH_2_ nanoparticles. Cyf (40 mg/mL) served as a suppressive control. Statistical analysis was performed by comparing cpm in particle-exposed cultures vs control cultures using Mann-Whitney test, *P* < 0.001.

No cytotoxic effect of the newly developed γ-Fe_2_O_3_&SiO_2_-NH_2_ core-shell particles on human peripheral blood cells was found when the cells were exposed to the whole range of particle concentrations (0.12-15 μg/cm^2^) during several time intervals (24, 48, and 72 h). No significant differences in basic proliferative activity of cells were observed (Figure 6).

Since no generally accepted nanoparticle positive and negative controls for immune assays are available at the moment, Cyf (well-known cytotoxic immunosuppressive agent) was used in three assays. They included assessment of cytotoxicity, proliferative activity of lymphocytes, and phagocytic activity of leukocytes. Appropriate doses were tested in previous experiments. Cyf (suppressive control) exposed to unstimulated peripheral blood cells for 48 and 72 h displayed a significant cytotoxicity.

**Figure 7:**
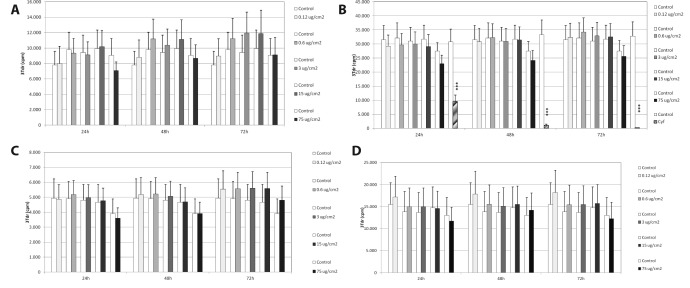
Proliferative response of human peripheral blood T-lymphocytes and T-dependent B-cells measured as incorporation of [^3^H]-thymidine (^3^Tdr) into replicating cells. Results are expressed as counts/min/culture (cpm). Bars indicate mean cpm group values (+ standard deviation) in cell cultures *in vitro* stimulated with (**A**) concanavalin, (**B**) phytohemagglutinin, (**C**) pokeweed mitogen, and (**D**) monoclonal anti-CD3 antigen. Blood cultures were treated with 0 (Control), 0.12, 0.6, 3, 15, and 75 μg of γ-Fe_2_O_3_&SiO_2_-NH_2_/cm^2^. The assay was performed after 24, 48, and 72 h of *in vitro* exposure of the peripheral blood cells (n = 10 human volunteers) to particles. Cyf (40 mg/mL) was used as a suppressive control. Statistical analysis was performed by comparing cpm in particle-exposed cell cultures vs control cultures using Mann-Whitney test. Significance: *** *P* < 0.001.

*In vitro* proliferative response of γ-Fe_2_O_3_&SiO_2_-NH_2_-treated human peripheral blood T-lymphocytes and T-dependent B-cells after 24, 48, and 72 h exposure was assessed by their stimulation with a panel of mitogens and CD3 antigen (Figures 7A-D). Incorporation of [^3^H]-thymidine into DNA of proliferating cells was highest ( ∼ 30 000 cpm) in cultures stimulated *in vitro* with PHA mitogen, as expected (Figure 7B). Statistical analysis revealed no significant effect of γ-Fe_2_O_3_&SiO_2_-NH_2_ particle concentration ranging 0.12-15 μg/cm^2^ on proliferative activity of lymphocytes stimulated with this mitogen in all time intervals. Moreover, regardless of the other mitogen and antigen used (Con A, PWM, and CD3), no significant changes in proliferative response of lymphocytes were found in cultures exposed to the whole concentration range of γ-Fe_2_O_3_&SiO_2_-NH_2_ particles (Figure 7A,C,D). A non-significant increase in proliferative response of T-cells stimulated through the T-cell receptor with CD3 antigen was found in the cell cultures treated with low doses of nanoparticles (0.12 µg/mL) for 24, 48, and 72 h (Figure 7D). Similarly, non-significant increase in proliferation was induced by middle concentrations of γ-Fe_2_O_3_&SiO_2_-NH_2_ particles (0.6-15 μg/cm^2^) in cell cultures stimulated with Con A (Figure 7A). Non-significant decrease was found also in cell cultures containing high dose of particles (75 µg/cm^2^) stimulated with PWM mitogen (Figure 7C). The effect of suppressive control (Cyf) on PHA-stimulated cell cultures was observed as a significant decrease in proliferative response of lymphocytes in all time intervals.

**Figure 8:**
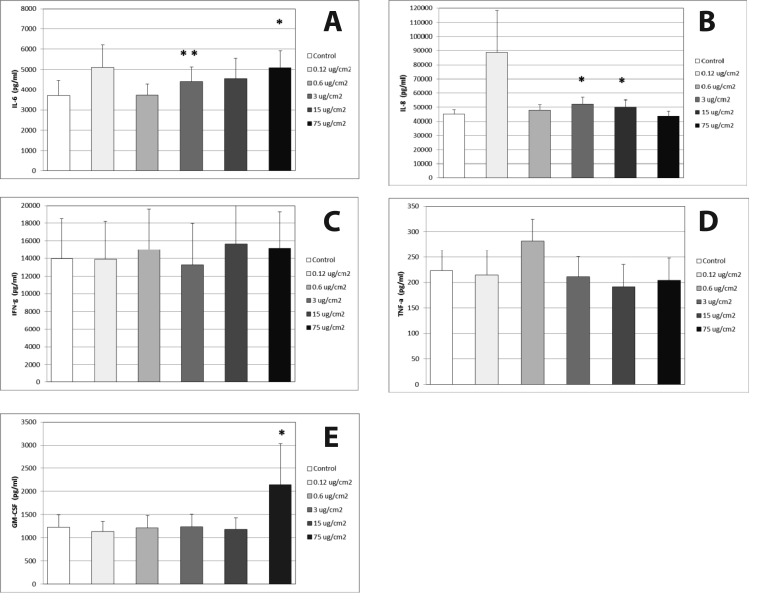
*In vitro* production of (**A**) interleukin-6, (**B**) interleukin-8, (**C**) interferon-gamma, (**D**) tumor necrosis factor-alpha, and (**E**) granulocyte macrophage colony stimulating factor cytokines in blood cells (n = 8 human volunteers) stimulated with phytohemagglutinin and for 48 h exposed to various concentrations of γ-Fe_2_O_3_&SiO_2_-NH_2_ nanoparticles: 0 (control), 0.12, 0.6, 3, 15, and 75 μg of γ-Fe_2_O_3_&SiO_2_-NH_2_/cm^2^. Results are expressed as pg/mL. Bars indicate mean group values (+ standard deviation). Statistical analysis was performed by comparing cytokine levels in particle-exposed and non-exposed (control) cell culture supernatants using paired *t* test. Significance: * *P* < 0.05, ** *P* < 0.01.

*In vitro production of cytokines IL-6, IL-8, IFN-γ, TNF-α, and GM-CSF*. *In vitro* production of cytokines IL-6, IL-8, TNF-α, IFN-γ, and GM-CSF in cells stimulated with PHA or PWM mitogens and treated for 48 h with whole range of particle concentrations is displayed on Figures 8 A-E. Significant increase in production of IL-6 without clear dose-dependence was found in cell cultures *in vitro* stimulated with PHA mitogen and exposed to middle and high particle doses (3 and 75 μg/cm^2^). Similarly, significantly enhanced production of IL-8 without clear dose-dependence was observed in cells *in vitro* simulated with the same mitogen (PHA) exposed to middle γ-Fe_2_O_3_&SiO_2_-NH_2_ doses (3 and 15 μg/cm^2^). On the other hand, production of IL-8 in PWM mitogen-stimulated cultures was significantly suppressed in almost all cells exposed to the particles. Levels of IFN-γ were significantly elevated in cells *in vitro* stimulated with PWM mitogen treated with two low γ-Fe_2_O_3_&SiO_2_-NH_2_ doses (0.12 and 0.6 μg/cm^2^). Regardless of the particle dose and mitogen used, production of TNF-α did not differ in all experiments. Production of GM-CSF cytokine was significantly increased in supernatants derived from cultures stimulated with PWM mitogen exposed to the high dose of γ-Fe_2_O_3_&SiO_2_-NH_2_ (75 μg/cm^2^).

**Figure 9:**
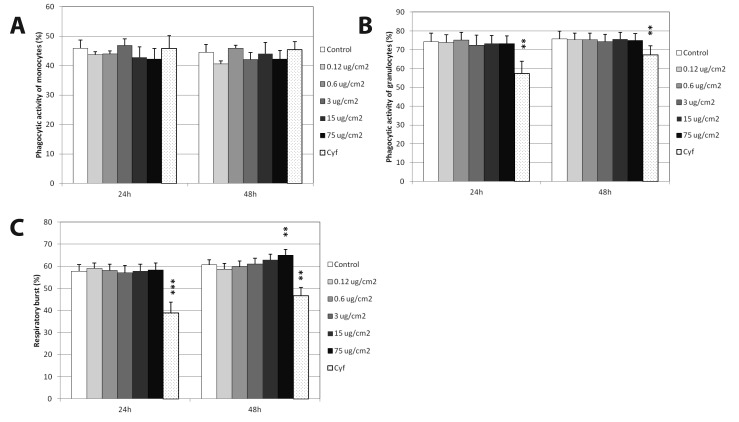
Phagocytic activity of (**A**) monocytes and (**B**) granulocytes evaluated by ingestion of fluorescein-labeled S*taphylococcus aureus,* and (**C**) respiratory burst of phagocytes monitored by flow cytometry using hydroethidine. Bars indicate mean group activity (+ standard deviation) in peripheral blood cultures treated *in vitro* with different concentrations of γ-Fe_2_O_3_&SiO_2_-NH_2_: 0 (control), 0.12, 0.6, 3, 15, and 75 μg/cm^2^; Cyf (5 mg/mL) served as a suppressive control. The assay was performed after 24 and 48 h of *in vitro* exposure of the peripheral blood cells (n = 10 human volunteers) to magnetic particles. Statistical analysis was performed by comparing phagocytic activity and respiratory burst in particle-containing samples with control using paired *t* test. Significance: ** *P* < 0.01, *** *P* < 0.001.

*Phagocytic activity and respiratory burst of leukocytes.* While the phagocytic activity was evaluated by ingestion of fluorescein-labeled S*taphylococcus aureus*, respiratory burst of phagocytes was measured using hydroethidine in the peripheral blood cells *in vitro* exposed to γ-Fe_2_O_3_&SiO_2_-NH_2_ for 24 and 48 h (Figures 9A-C). No interference of particles with the assay was observed. Phagocytic activity of monocytes in γ-Fe_2_O_3_&SiO_2_-NH_2_-treated cell cultures did not show significant changes compared to the control cultures after both 24 and 48 h exposure (Figure 9A). A similar situation was observed also for the granulocytes (Figure 9B). Respiratory burst of phagocytes increased with increasing γ-Fe_2_O_3_&SiO_2_-NH_2_ concentration and was significantly enhanced after exposure to high dose of particles (75 µg/cm^2^) after 48 h exposure. Suppressive control agent (Cyf) significantly decreased phagocytic activity of granulocytes and respiratory burst of phagocytes in both time exposure intervals (24 and 48 h; Figure 9B,C).

## Discussion

The core-shell γ-Fe_2_O_3_&SiO_2_-NH_2_ nanoparticles were prepared by chemical coprecipitation, which was followed by oxidation and silanization. The coprecipitation technique belongs to the most convenient methods for production of iron oxide particles, which find many biological applications, such as in cell imaging and tracking, drug and gene delivery, hyperthermia, and capture of various cells and biomolecules (21). Morphology including shape, size, and its distribution depend on various reaction parameters, eg, type of salt, Fe(II)/Fe(III) ratio, temperature, pH, ionic strength, stirring, etc. Big advantage of these particles consists in their superparamagnetic behavior, which means that below certain size (<20 nm) the particles have no hysteresis and are attracted by a magnet, however, they are easily redispersed in water when the magnetic field is removed (22). In this paper, optimal reaction conditions led to formation of 9 nm γ-Fe_2_O_3_ particles. After their modification with silica and aminosilica, diameter of the particles increased to 13 nm. Properties of the starting and modified particles were determined by a range of physico-chemical methods including TEM, DLS, and XPS measurements. The coating on the iron oxide nanoparticle surface was confirmed by TEM, ATR FTIR spectroscopy, and XPS analysis. Silica coating of the γ-Fe_2_O_3_ prevented aggregation of the particles in water and enhanced their chemical stability to several months.

In the biological *in vitro* experiments, the cytotoxicity and the effect of particles on the immune response were examined. Five different particle concentrations, which are typically used in analogous works, were selected ranging 0.12-75 µg/cm^2^, which corresponded to 0.17-106 μg of particles/mL (calculated Fe content was from 21.25 ng/mL to 13.25 μg/mL). In our experimental setup, γ-Fe_2_O_3_&SiO_2_-NH_2_ particles displayed no cytotoxic effect on human peripheral blood cells. The newly synthesized particles can be therefore considered as a nanomaterial with very low *in vitro* cytotoxicity. However, *in vivo* experiments will be needed to confirm the results. Although *in vitro* and *in vivo* comparison of particle doses is very inaccurate even almost impossible; in human clinical diagnostics, the recommended dose for liver imaging is 15 µmol of Fe per kg if dextran-coated iron oxide (Endorem) is used.

With the aim to gain an overview on concentrations required for MR imaging, capacity of human monocytes to phagocyte iron oxide of different particle sizes, concentrations, and incubation times was investigated (23). The intracellular iron content was measured by atomic emission absorption spectrometry. A significantly higher cellular iron oxide uptake was found after incubation with large compared to small particles. It means that the former particles were better suited for MRI than the latter ones for *ex vivo* labeling of human monocytes prior to injection (23).

Function of lymphocytes was examined in cultures derived from human peripheral blood and *in vitro* treated either with stimulators of T-cells (PHA, Con A mitogens, and CD3 antigen) or stimulator of T-dependent B-cell response (PWM mitogen). In mitogen-stimulated cultures, differences in proliferative activity between the particle-exposed and unexposed lymphocytes might become more visible. This can be attributed to the increased sensitivity of the assay due to higher counts per minute per culture. Nevertheless, no significant differences in proliferative response of T-lymphocytes, as well as T-dependent B-cells, treated with all γ-Fe_2_O_3_&SiO_2_-NH_2_ concentrations compared to the control untreated cells were found at all time intervals.

Significantly increased production of IL-6, IL-8, and GM-CSF cytokines by human blood cells treated with middle and high doses of γ-Fe_2_O_3_&SiO_2_-NH_2_ particles (3, 15, and 75 µg/cm^2^) were observed in cell cultures stimulated with PHA mitogen. However, elevated productions were without clear dose-dependence. This phenomenon can be partially explained by different nanoparticle dispersibility in the wells resulting in partial agglomeration and variable nanoparticle/cell ratios. Absence of γ-Fe_2_O_3_&SiO_2_-NH_2_ dose dependence in stimulation of IL-6, IL-8, and GM-CSF production has to be taken into consideration in future investigations. IL-6 is a pro-inflammatory cytokine secreted by T-cells, which stimulates immune response, eg, during infection and after tissue damage leading to inflammation. IL-6 is known to stimulate the inflammatory and auto-immune processes in many diseases, such as diabetes or atherosclerosis. IL-8 is a chemokine produced by macrophages and other cell types. IL-8 has two primary functions: induction of phagocytosis and chemotaxis, primarily neutrophils, but also other granulocytes causing their migration toward the site of infection. GM-CSF is a white blood cell growth factor, which stimulates stem cells to produce granulocytes and monocytes. GM-CSF is part of the immune/inflammatory cascade, by which activation of small number of macrophages can rapidly lead to an increase in their numbers for fighting infection (24).

Results of the effect of γ-Fe_2_O_3_&SiO_2_-NH_2_ particles on phagocytic activity and respiratory burst of phagocytes in human peripheral blood cultures are in agreement with studies on the interactions of Ferumoxtran-10 (F-10) iron oxide with human monocyte-macrophages *in vitro*, where lack of pro-inflammatory activity were assessed (25). After 72 h incubation, F-10 (1 mg/mL) was not toxic and only mildly toxic at high concentrations (10 mg/mL). Viability of the cells was not affected during 14 days. F-10 did not stimulate cytokines (interleukin-12, interleukin-6, tumor necrosis factor-α, and interleukin-1β), superoxide anion production, or Fc-receptor-mediated phagocytosis. Similarly, amino-poly(vinyl alcohol)-coated magnetic particles did not affect viability of human immune cells, but cytokine secretion (26). At the same time, percentage of viable macrophages increased, especially when the particles were added very early in the differentiation process.

On the other hand, magnetic nanoparticles induced formation of membranous ferroportin and incited secretion of ferritin, TNF-α, and IL-10 in human histiocytic lymphoma cells (U937) and human monocyte leukemia cells without any decrease of cell viability (27). A dose- and time-dependent cytotoxicity increase of oleate- or oleate/poly(ethylene glycol)/poly(lactic-*co*-glycolic acid)-coated magnetic particles was found in human lung adenocarcinoma epithelial (A549) and human embryo lung cells (HEL 12469) (28).

Animal experiments showed that iron oxide particles administered in mice in successive intratracheal instillations modulated the pulmonary immune response to ovalbumin (OVA) depending on the particle dose and size. At high and intermediate doses (4 × 250 or 4 × 500 μg of nanoparticles/mouse), the OVA-induced allergic response was significantly inhibited, as evidenced by the decrease in eosinophil cell influx and specific IgE levels. However, the low dose (4 × 100 μg of nanoparticles/mouse) had no significant effect on the OVA allergic response, while the same nanoparticle dose had an adjuvant effect on the Th2 response to OVA (29).

In conclusion, superparamagnetic γ-Fe_2_O_3_ nanoparticles were successfully developed and modified with two silica precursors. Assessment of interaction of γ-Fe_2_O_3_&SiO_2_-NH_2_ nanoparticles with human peripheral blood cells using TEM showed that the particles were localized within the cytoplasm of treated cells and in the extracellular space. No preferential localization in the cells was observed. The γ-Fe_2_O_3_&SiO_2_-NH_2_ particles proved to be non-toxic even at high dose (75 µg/cm^2^) and after long-time incubation period (72 h). No significant differences in proliferative response of T-lymphocytes, as well as T-dependent B-cells treated with γ-Fe_2_O_3_&SiO_2_-NH_2_ particles in all concentrations and time exposures were found. Significantly increased production of IL-6, IL-8, and GM-CSF cytokines by human blood cells treated with middle and high doses of γ-Fe_2_O_3_&SiO_2_-NH_2_ particles (3, 15, and 75 µg/cm^2^) was observed without clear dose-dependence. No significant changes in production of TNF-α and IFN-γ was observed. Magnetic nanoparticles did not affect phagocytic activity of monocytes and granulocytes. Respiratory burst of phagocytes was significantly enhanced in cell cultures exposed to high particle dose (75 µg/cm^2^) for 48 h. Cytotoxicity and *in vitro* immunotoxicity of new porous γ-Fe_2_O_3_&SiO_2_-NH_2_ core-shell nanoparticles were minimal, however, more assessments will be needed before possible use in human medicine, eg, in cell labeling, MRI, and drug delivery.
